# Supplementary material for: Contemporary high resolution European forest structure assessed using tree-level National Forest Inventory data
Source: PLoS One. 2026 Jun 5;21(6):e0346611. doi: 10.1371/journal.pone.0346611 (PMC13240908; doi:10.1371/journal.pone.0346611)
Supplement: S1 File — (DOCX) [file pone.0346611.s001.docx]

# Supplementary material

# S1. Country results

Spain emerged as the country with the most significant representation of the Single-species Regular class, with over 79% of all NFI plots falling into this category (Table S1). Ireland also exhibited a substantial share of SSR, comprising approximately 69%, followed closely by Poland, Luxembourg, Norway and Belgium, each with around 60% of plots categorized under the SSR class. In contrast, the other two classes dominated by a single-species constituted only a small fraction of all plots across countries, rarely exceeding 3%.

France stood out as the country with the most diverse forest structure, where the MSR class accounted for 39.6% of all plots, MSL for 12%, and Multiple-species Irregular for about 2%. Switzerland held the highest share of the MSI class, with 3.7% of plots falling into this category, while MSL represented 7.3%, and MSR constituted more than 47% of the plots.

France had the highest prevalence of the MSL class, with 12% of plots falling into this category, followed by Flanders with 10.6% and Netherlands and Switzerland with respectively 8 and 7.3%.

Table S1. Percentages of plots by each forest structure class per country. *Acronyms: Single-species irregular (SSI), Single-species admixture (SSA), Single-species regular (SSR), Multiple-species irregular (MSI), Multiple-species layered (MSL), Multiple-species regular (MSR).*

| **Country** | **SSI (%)** | **SSA (%)** | **SSR (%)** | **MSI (%)** | **MSL (%)** | **MSR (%)** |
| --- | --- | --- | --- | --- | --- | --- |
| Austria | 0.0 | 0.0 | 57.4 | 0.0 | 0.0 | 42.6 |
| Flanders | 1.2 | 2.9 | 51.2 | 1.6 | 10.6 | 32.5 |
| Wallonia | 0.7 | 0.8 | 64.6 | 0.0 | 1.3 | 32.6 |
| Belgium ^^[[1]](#footnote-1)^^ | 0.8 | 1.2 | 61.9 | 0.3 | 3.2 | 32.6 |
| Croatia | 0.0 | 0.2 | 58.5 | 0.0 | 0.1 | 41.2 |
| Czechia | 1.2 | 1.4 | 48.2 | 1.7 | 5.3 | 42.2 |
| Denmark | 2.0 | 1.6 | 47.6 | 1.6 | 5.5 | 41.7 |
| Finland | 0.2 | 0.2 | 56.7 | 0.2 | 0.7 | 42.0 |
| France | 2.1 | 2.6 | 42.3 | 2.1 | 11.7 | 39.2 |
| Germany | 1.4 | 0.5 | 52.3 | 1.2 | 4.6 | 40.0 |
| Ireland | 0.3 | 0.4 | 68.7 | 1.0 | 3.5 | 26.0 |
| Italy | 3.5 | 2.2 | 43.6 | 2.3 | 6.3 | 42.1 |
| Luxembourg | 0.0 | 0.0 | 63.2 | 0.0 | 0.0 | 36.8 |
| The Netherlands | 1.3 | 3.1 | 47.7 | 1.0 | 8.0 | 38.9 |
| Norway | 0.6 | 1.1 | 62.4 | 0.3 | 2.0 | 33.7 |
| Poland | 0.8 | 1.4 | 63.3 | 0.6 | 3.7 | 30.2 |
| Slovakia | 2.8 | 1.1 | 48.5 | 1.5 | 3.3 | 43.0 |
| Spain | 0.0 | 0.0 | 79.7 | 0.0 | 0.0 | 20.2 |
| Sweden | 0.5 | 0.4 | 52.4 | 0.3 | 1.6 | 44.8 |
| Switzerland | 3.8 | 1.7 | 35.8 | 3.7 | 7.3 | 47.7 |
| Total for 18 countries | 0.9 | 0.9 | 56.4 | 0.8 | 3.3 | 37.7 |

#

1. weighed on the areas of Walloon and Flanders [↑](#footnote-ref-1)
