# Supplementary material for: Contemporary high resolution European forest structure assessed using tree-level National Forest Inventory data
Source: PLoS One. 2026 Jun 5;21(6):e0346611. doi: 10.1371/journal.pone.0346611 (PMC13240908; doi:10.1371/journal.pone.0346611)
Supplement: S2 File — (DOCX) [file pone.0346611.s002.docx]

# S2. Method comparison

The Danish data included the categories group-cohort forests (GCF), one storey (1S), two storey (2S), three storey (3S) and plenterwald (PW), the Dutch data included the categories even-aged (EA) and uneven-aged (UA) forests, while the Swiss data contained the categories single-layered (SL), multi-layered (ML), stratified (ST) and clustered (CL). To provide a visual assessment of this contingency, a matrix plot was generated for the Pearson residuals. The Chi-squared test of independence was used to quantify the statistical significance of the relationship between the two groups. The test is usually applied on contingency tables to test for independence between rows and columns [89] and is one of the most common statistical analyses in evaluation and research [90] . Additionally, the strength of the relationship between the assessed categories and the field-measured categories was evaluated using Cramer's V.

The comparison between the forest structure categories assessed through our method and those measured in the field was conducted on a plot-by-plot basis. The Chi-squaredSquare test results reveal significant relationship between the assumed classes outlined in this study and the measured classes in the field, with very low p-values (**Table** S2). Cramer’s V values range from 0.237 for Switzerland to 0.361 for Denmark, close to or above the 0.25 threshold as outlined by [91]. We can thus conclude that there is a moderate to good relationship between our classes and the country-specific category groups for all three countries.

**Table S2**. The p-values and Cramer’s V for the Chi-squared tests

| Country | Chi-squares Test p-Value | Cramer's V |
| --- | --- | --- |
| Denmark | 2.090624e^-18^ | 0.3607488 |
| Netherlands | 2.980213e^-40^ | 0.3440803 |
| Switzerland | 2.797223e^-41^ | 0.2373362 |

In the context of Denmark, the two irregular classes, SSI and MSI, exhibited a robust negative correlation with the GCF and 1S classes, while demonstrating a pronounced positive correlation with 2S, PW, and notably, 3S, underscoring a highly significant relationship among these categories. The MSL class displayed the most substantial positive correlation with 2S and a negative correlation with 1S, whereas SSA showed the highest positive correlation with PW and the most negative correlation with 1S. Noteworthy is the strong positive correlation between SSR and 1S, contrasted with MSR's negative correlation with this class but positive correlations with PW and 2S, warranting further investigation and discussion.


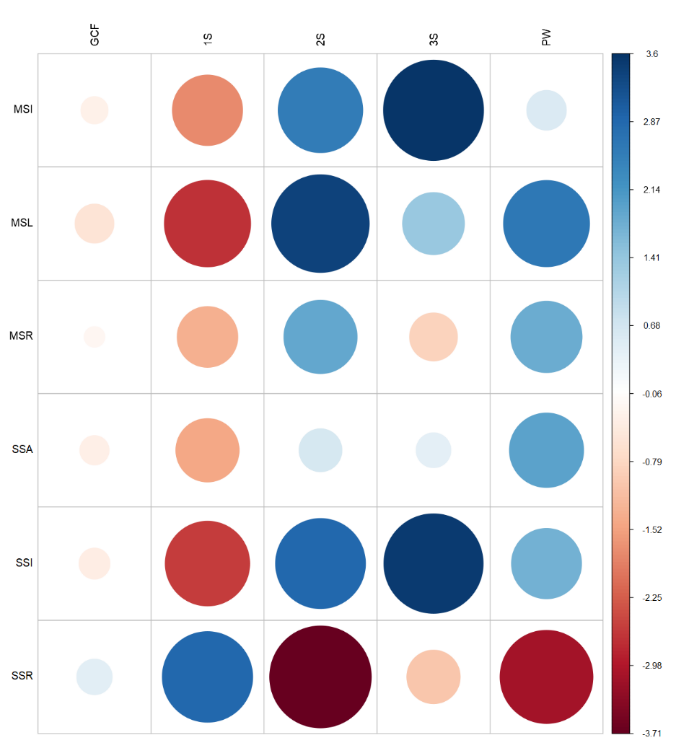


*Figure S1 . Correlation matrix plot for the assessed and measured structure categories for Denmark. Acronyms of this study’ classes: Single-species irregular (SSI), Single-species admixture (SSA), Single-species regular (SSR), Multiple-species irregular (MSI), Multiple-species layered (MSL), Multiple-species regular (MSR). Positive and negative correlations are given by the colors, the size of the ball indicates the number of plots. See for explanation of the Danish classes the S4.*

Similar to Denmark, the Netherlands exhibits a parallel trend. The irregular classes MSI and SSI, as well as SSA, and MSL, demonstrated positive correlations with the UA (unevenaged) class observed in the field and negative correlations with EA (evenaged), with MSL displaying the highest positive and lowest negative correlation. Conversely, the SSR class displays the highest positive correlation with EA and the lowest with UA, while MSR demonstrates the opposite trend, with a positive correlation with UA and a negative one with EA, prompting further discussion.


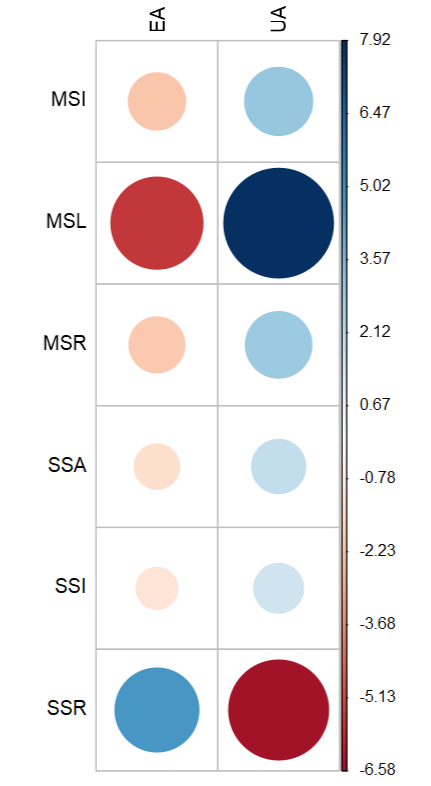


*Figure S2* ***.*** *Correlation matrix plot for the assessed and measured structure categories for Netherlands (evenaged (EA), unevenaged (UA)). Acronyms of this study’ classes: Single-species irregular (SSI), Single-species admixture (SSA), Single-species regular (SSR), Multiple-species irregular (MSI), Multiple-species layered (MSL), Multiple-species regular (MSR). Positive and negative correlations are given by the colors, the size of the ball indicates the number of plots.*

Just like the patterns observed in the two preceding countries, Switzerland exhibits the same trend, as depicted in Figure 8. The two irregular classes, MSI and SSI, displayed a strong positive correlation with the ST category and a negative correlation with SL. Conversely, SSR demonstrated the highest positive correlation with SL and a negative correlation with ML. Similarly, in line with the previous examples of Denmark and the Netherlands, the MSR category exhibited a positive correlation with ML and a negative correlation with SL, mirroring the trend observed in the MSL category.

 
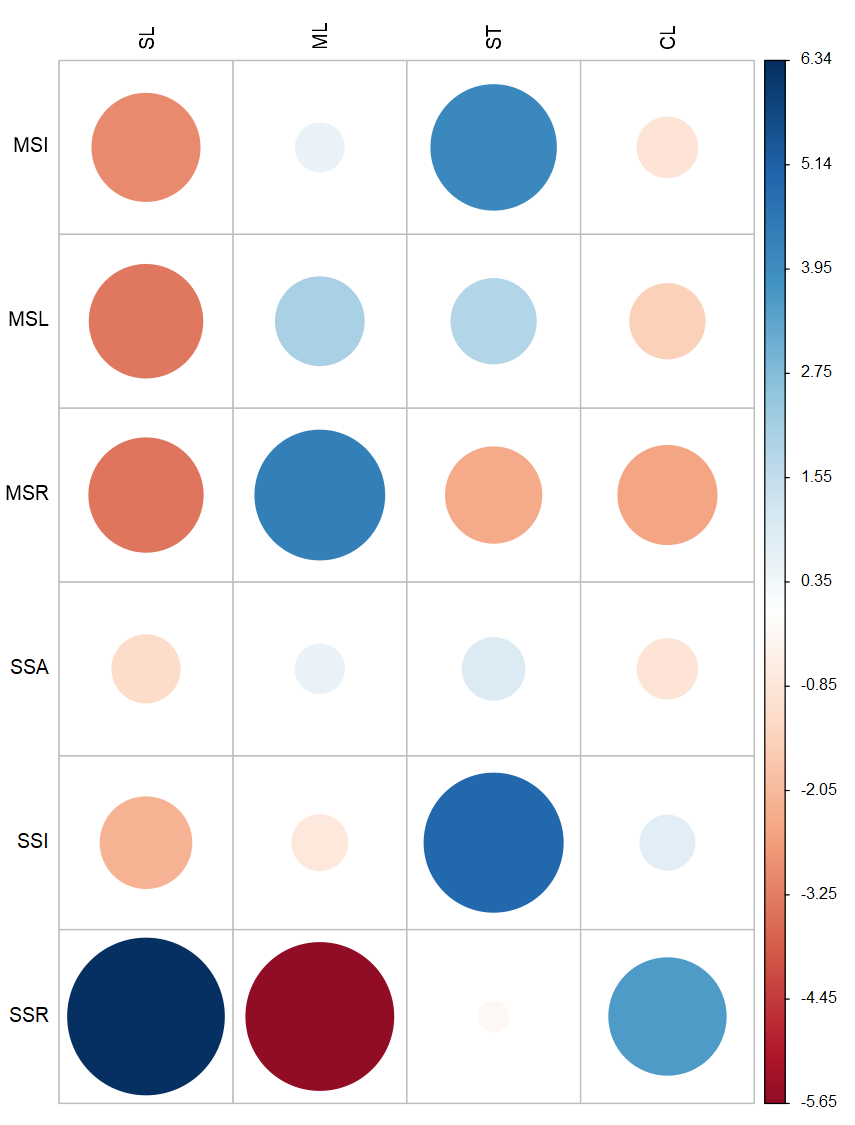


*Figure S3****.*** *Correlation matrix plot for the assessed and measured structure categories for Switzerland (single-layered (SL), multi-layered (ML), stratified (ST) and clustered (CL).). Acronyms of this study’ classes: Single-species irregular (SSI), Single-species admixture (SSA), Single-species regular (SSR), Multiple-species irregular (MSI), Multiple-species layered (MSL), Multiple-species regular (MSR). Positive and negative correlations are given by the colors, the size of the ball indicates the number of plots.*
