# Supplementary material for: Contemporary high resolution European forest structure assessed using tree-level National Forest Inventory data
Source: PLoS One. 2026 Jun 5;21(6):e0346611. doi: 10.1371/journal.pone.0346611 (PMC13240908; doi:10.1371/journal.pone.0346611)

# S3. Criteria employed in the field in the NFIs of Denmark, Netherlands, Switzerland by which structure is assessed (see sources in Table 1)

## Netherlands:

From the field manual :

A forest (stand = plot) is evenaged when the cover of the understorey or the medium storey forms less than 50%. Or the basal area share of the medium storey is less than < 20% of total basal area

A forest (stand = plot) is unevenaged when the cover of the under- or medium storey (Dbh> 5 cm) is more than 50%. Or the basal area share of the under- or mediumstorey >20% and the difference in age of the understorey at least 20 years.

## Denmark

In Danish NFI the field crew judges the stand as follows. The Danish inventory manual does not give clear criteria

1: Én-etageret (one crown layer)

2: To-etageret (two crown layers)

3: Tre-etageret (three crown layers)

4: Gruppevis alderstruktureret (group-cohort forests (GCF))

5: Plukhugstagtig aldersstruktureret ( plenterwald (PW)).

## Switzerland

The swiss NFI distinguish: single-layered (einschichtig, SL), multi-layered (mehrschichtig, ML), stratified (stufig, ST) and clustered (Rottenstruktur, CL).

See below snapshot from Schweizerisches Landesforstinventar Feldaufnahme-Anleitung 2017 [92]. Reproduced with permission


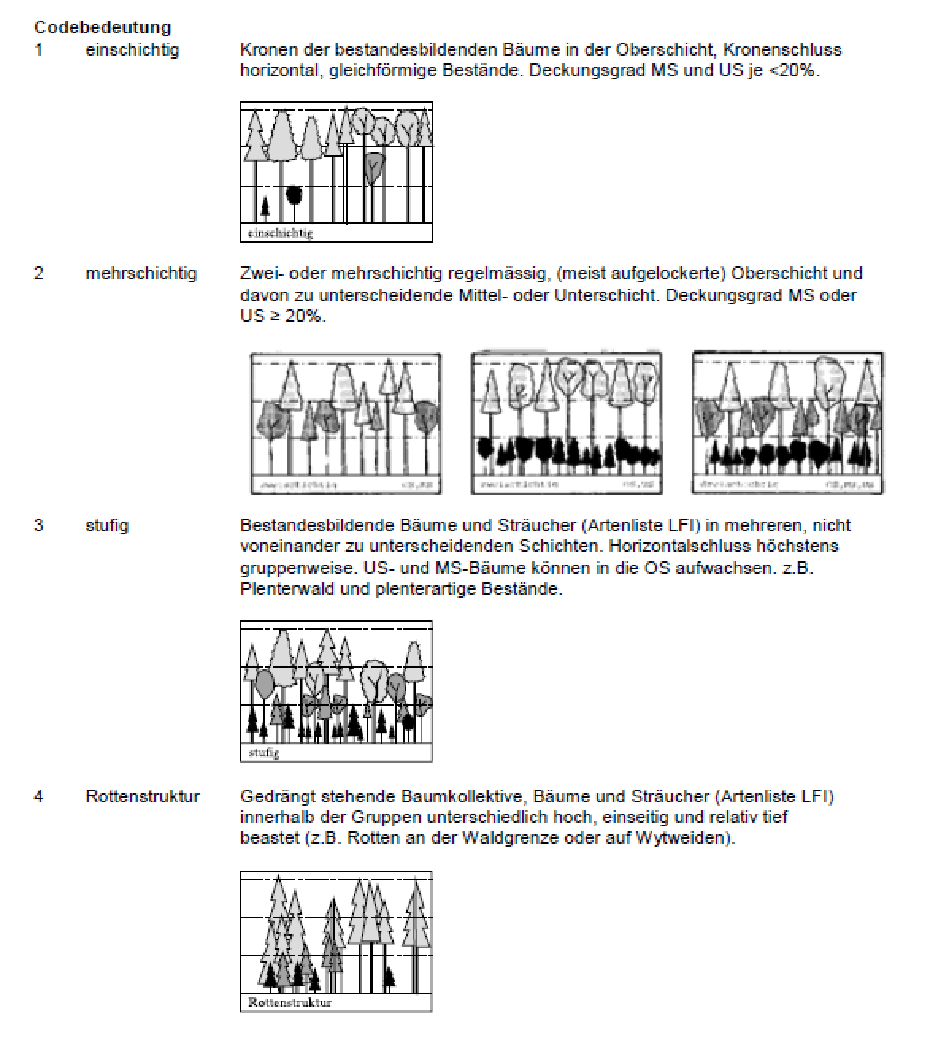

Supplement: S3 File — (DOCX) [file pone.0346611.s003.docx]
