# Supplementary material for: Contemporary high resolution European forest structure assessed using tree-level National Forest Inventory data
Source: PLoS One. 2026 Jun 5;21(6):e0346611. doi: 10.1371/journal.pone.0346611 (PMC13240908; doi:10.1371/journal.pone.0346611)
Supplement: S4 File — (DOCX) [file pone.0346611.s004.docx]

S4. stand level example analysis of Gini for three types of stands For the two layered stand, the bottom graph also gives the Gini excluding the 5-10cm trees.
