# Supplementary material for: Contemporary high resolution European forest structure assessed using tree-level National Forest Inventory data
Source: PLoS One. 2026 Jun 5;21(6):e0346611. doi: 10.1371/journal.pone.0346611 (PMC13240908; doi:10.1371/journal.pone.0346611)

# S5. National level example results with including and excluding the 5-10 cm class

On the y axis the number of plots that falls in a class. The two regular classes gain in prominence in case the 5-10 cm class is excluded.


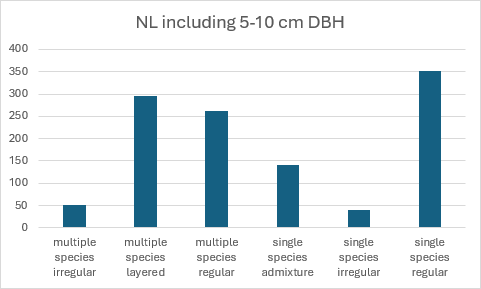

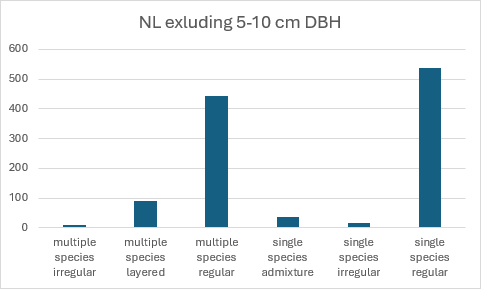

Supplement: S5 File — (DOCX) [file pone.0346611.s005.docx]
